# Loeffler's Endocarditis and the Diagnostic Utility of Multimodality Imaging

**DOI:** 10.7759/cureus.10061

**Published:** 2020-08-26

**Authors:** Saira Afzal, Taha Ahmed, Talha Saleem, Albert Chan

**Affiliations:** 1 Internal Medicine, Cleveland Clinic Fairview Hospital, Cleveland, USA; 2 Internal Medicine, Cleveland Clinic Foundation, Cleveland, USA; 3 Cardiovascular Medicine, Cleveland Clinic Fairview Hospital, Cleveland, USA

**Keywords:** transthoracic echocardiogram, loeffler’s endocarditis, cardiac mri, eosinophilia

## Abstract

Loeffler’s endocarditis is a rare form of restrictive cardiomyopathy associated with eosinophilia and endomyocardial fibrosis. It manifests most commonly as diastolic dysfunction or valvular abnormalities due to eosinophilic infiltration and degranulation. Herein, we chronicle a case of left ventricular involvement with Loeffler’s endocarditis. We emphasize the utility of multimodality imaging including two-dimensional echocardiography and cardiac magnetic resonance imaging in providing diagnostic information, as there is no standardized diagnostic criteria to date.

## Introduction

Loeffler's endocarditis was first described by Wilhelm Loeffler in 1936 who called it “fibroplastic parietal endocarditis with blood eosinophilia”. It is a rare form of restrictive cardiomyopathy that results from endomyocardial infiltration of eosinophils resulting in fibrosis. Loeffler's endocarditis is normally considered as a cardiac manifestation of hypereosinophilic syndrome (HES). HES is defined as persistent eosinophilia of 1,500 eosinophils/mm^3^ for longer than six months and lack of evidence of parasitic, allergic, and other known causes of eosinophilia [1]. Here we describe a case of a 66-year-old female who was diagnosed with Loeffler’s endocarditis, but her eosinophil count was not high enough to be characterized as having HES. This case demonstrates the use of cardiac magnetic resonance imaging (CMR) as a noninvasive and reliable tool to diagnose Loeffler’s endocarditis.

## Case presentation

The patient is a 66-year-old female with a past medical history significant of asthma and chronic obstructive pulmonary disease (COPD) overlap syndrome, allergic rhinitis, hypothyroidism, diabetes mellitus type II, remote history of unprovoked deep venous thrombosis, anxiety/bipolar disorder, and back pain presented to our hospital with persistent exertional shortness of breath. Her prior cardiac workup included a transthoracic echocardiogram five years prior that showed stage 2 left ventricular (LV) diastolic dysfunction with an ejection fraction (EF) of 63%. A review of her medical records showed that she had two recent admissions with a similar complaint, and her symptoms were considered as COPD exacerbation and she was treated with antibiotics and steroids. In this presentation, the patient was hemodynamically stable. Physical exam was done showing positive jugular venous pulsations, normal heart sounds with no heaves, rub, or murmur. Laboratory findings were remarkable for leukocytosis; however, patients recently completed a course of steroids for COPD. The patient also had eosinophilia of 0.99 k/microL (normal <0.46 k/microL). The patient previous labs showed an intermittent elevation in eosinophils with maximum eosinophilia of 1.13 k/microL in the past. Her brain natriuretic peptide was elevated to 3,197 pg/mL (normal < 100 pg/mL). A chest X-ray was normal. EKG showed no ischemic changes with known left bundle branch block. Transthoracic echocardiogram (TTE) was done showing preserved EF with moderate mitral regurgitation. A flat filling defect was seen in the cardiac apex with the use of definity contrast (Video 1).

**Video 1:** Transthoracic echocardiogram (TTE) with definity contrast TTE showing flat filling defect in the left ventricular apex

**Figure 1:**
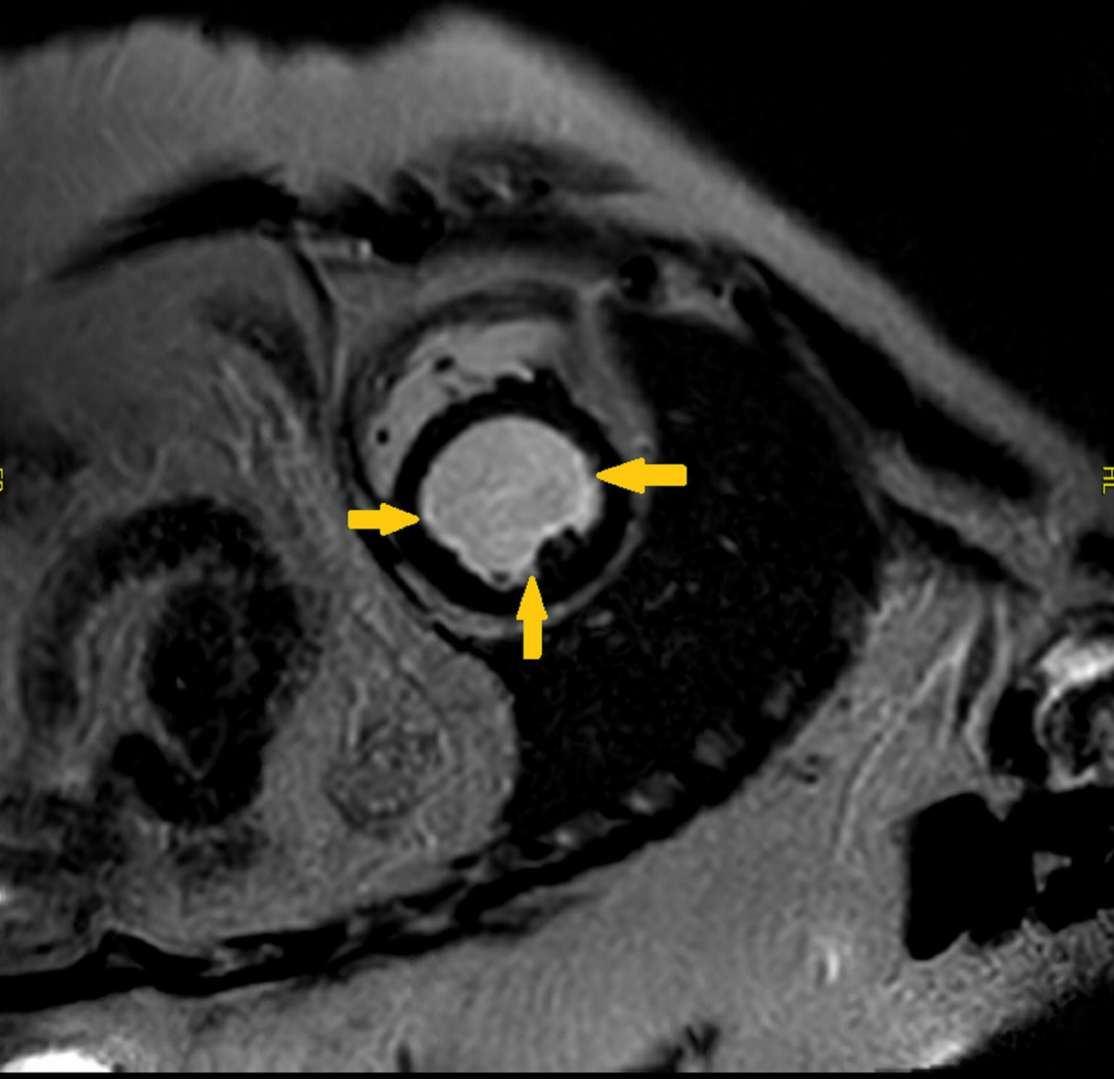
Cardiac MRI Cardiac MRI in short axis view showing rim of subendocardial delayed enhancement (arrows)

Due to concern for Loeffler’s cardiomyopathy, CMR was ordered. MRI showed rim of subendocardial delayed enhancement involving the apical segments and extending into the mid-walls (Figure 1).

**Figure 2:**
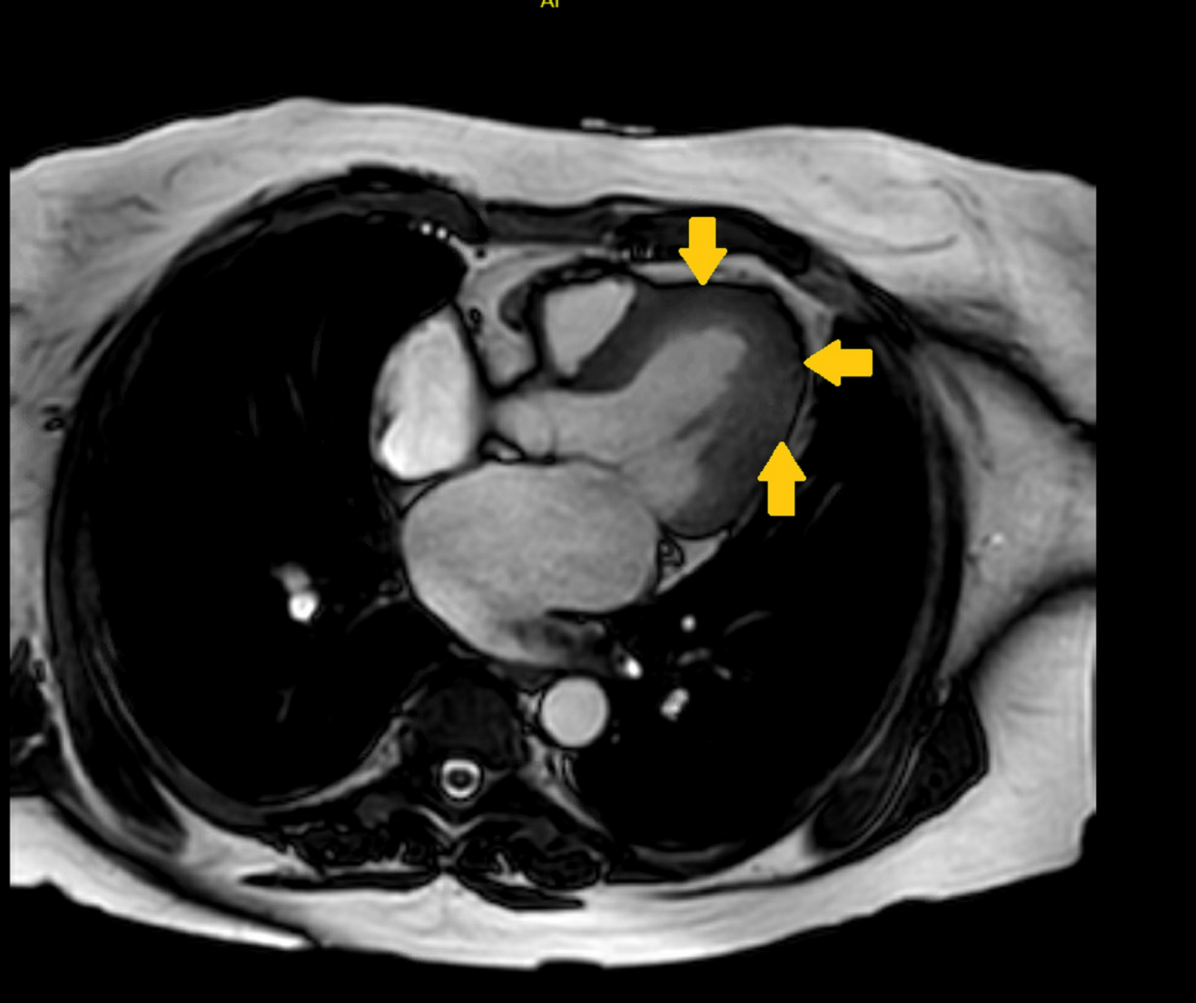
Cardiac MRI Cardiac MRI axial view showing low subendocardial signal in the left ventricular apical segment (arrows)

**Figure 3:**
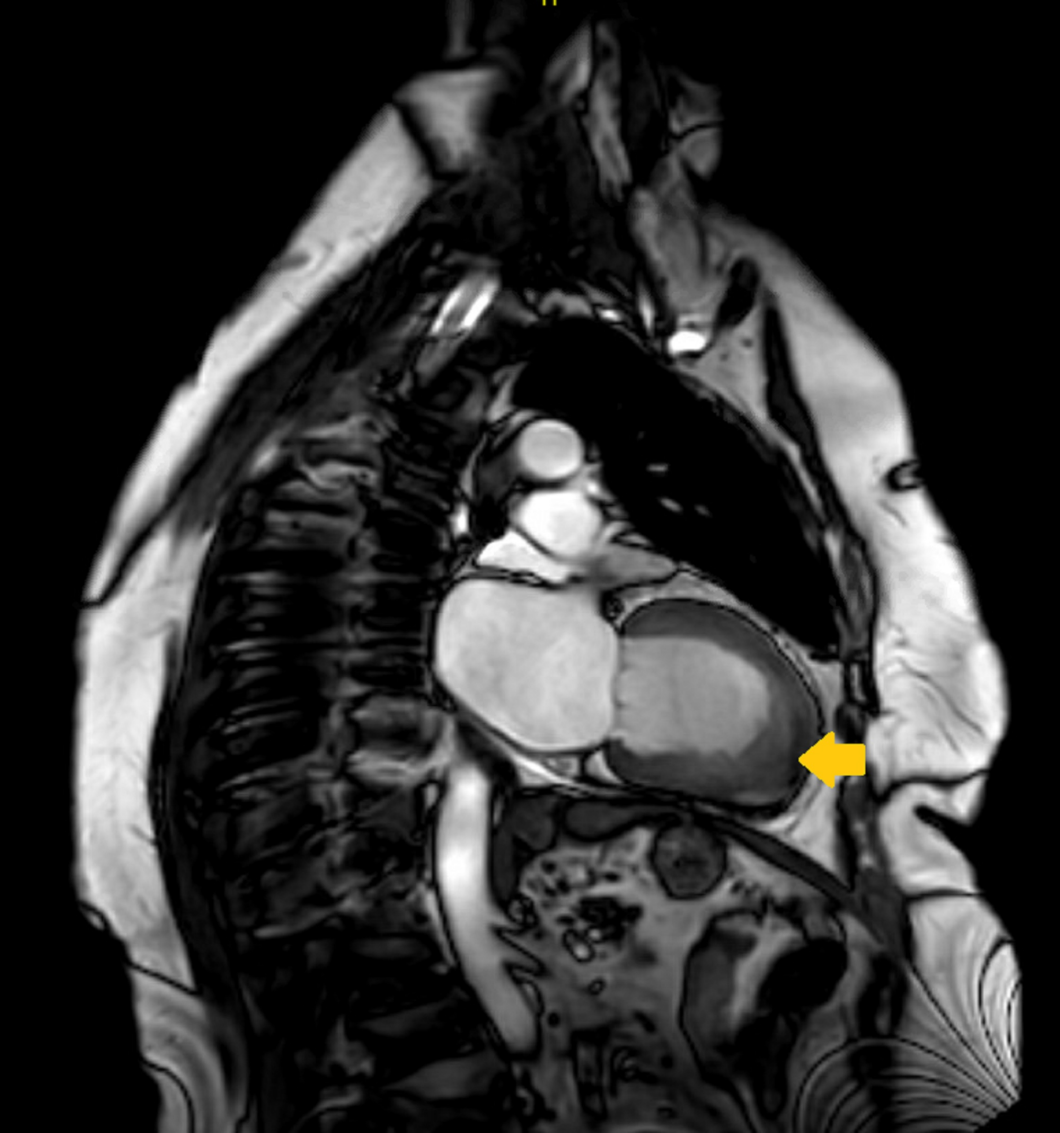
Cardiac MRI Cardiac MRI saggital view showing low subendocardial signal in the left ventricular apical segment (arrow)

It corresponded to low subendocardial signals within these territories on cine images (Figures 2, 3).

**Figure 4:**
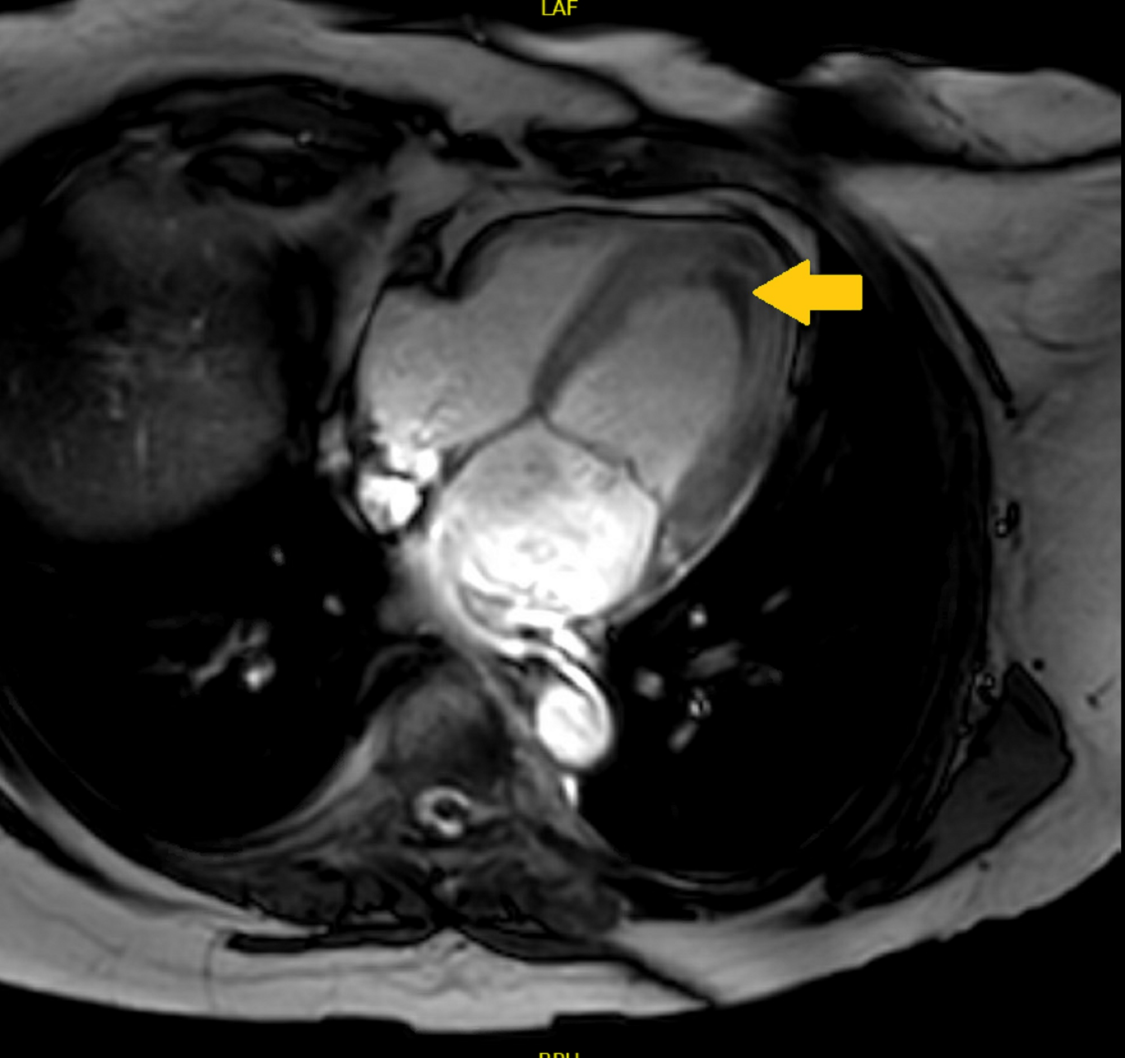
Cardiac MRI Cardiac MRI axial view showing well-defined area of low signal intensity consistent with thrombus formation (arrow)

The appearance was consistent with endocardial fibrosis/inflammation typical for Loeffler’s cardiomyopathy. A well-defined area of low signal intensity, consistent with thrombus on tissue characterization, was also seen along with mitral valve regurgitation (Figure 4).

The patient was started on warfarin for anticoagulation. The patient's eosinophilia did not meet the criteria for primary HES; ruling out other causes, it was attributed to some of her medical issues of rhinosinusitis and asthma. Further testing including bone marrow biopsy and molecular testing was not done, and in the absence of hypereosinophilia, the patient was not started on steroids.

**Figure 5:**
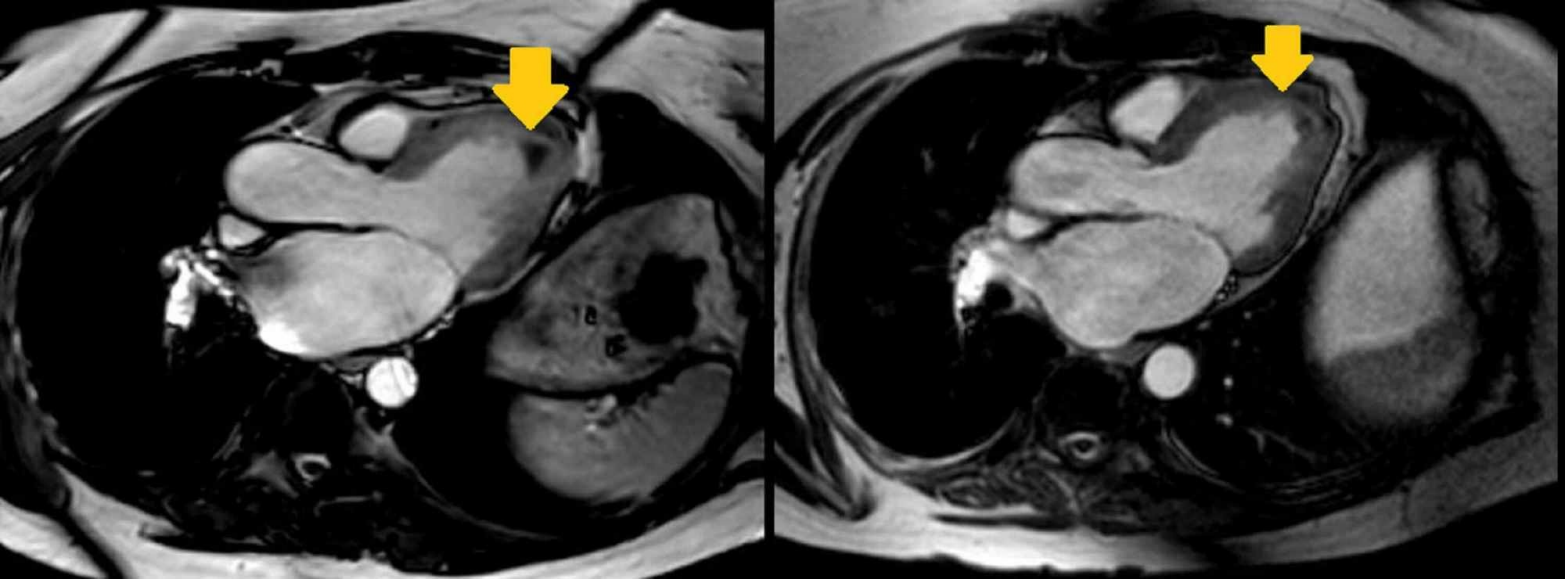
Cardiac MRI (before and after) Cardiac MRI four months later showing improvement in the hypoattenuation of the left ventricular apex (arrows indicating comparison)

Repeat TTE after four months showed normal EF with grade I diastolic function. LV apical wall appeared less echo dense as compared to before. Repeat cardiac MRI was done showing that the hypoattenuation in the apex has significantly improved with resolution of the thrombus (Figure 5).

Delayed enhancement of subendocardial tissue was still present.

## Discussion

Loeffler's endocarditis is commonly characterized as a rare complication of HES. However, any condition causing an eosinophilic state can make patients susceptible to this condition. Eosinophil-associated cardiac injury is described as having three stages. The first stage is associated with eosinophilic infiltration resulting in the formation of toxic cationic proteins causing acute necrosis, this stage is mostly asymptomatic. The second stage is associated with thrombus formation. The final stage is characterized by fibrosis resulting in restrictive cardiomyopathy [2,3]. Patients with eosinophil-associated endocarditis can present with a wide array of symptoms, including dyspnea, chest pain, cough, congestive heart failure, and valvular involvement. The most involved cardiac valve is the mitral valve resulting in mitral valve insufficiency in almost 42% of cases. It can also result in involvement if aortic valve causing aortic valve stenosis and regurgitation in 4% of cases [3]. Diagnosis usually requires endomyocardial biopsy that is considered as a gold standard; however, it is an invasive test and the diseased area can be missed resulting in false-negative biopsy results. Noninvasive diagnostic tools to assist in diagnosis include electrocardiography and echocardiography. CMR has emerged as an extremely useful device to help in the diagnosis of Loeffler’s endocarditis. Loeffler's endocarditis does not show any specific pathognomonic findings on EKG. EKG may show T-wave inversions, left atrial enlargement, LV hypertrophy, incomplete right bundle branch block, and left axis deviation. Echocardiography findings suggestive of Loeffler’s endocarditis include endomyocardial thickening, apical thrombus, LV hypertrophy, and involvement of mitral valve cusp causing mitral valve regurgitation, consistent with the findings in our case [4]. CMR can identify injured myocardial tissue and differentiate between inflammation and fibrosis by analyzing the difference between early and late contrast enhancement. Late contrast gadolinium enhancement is pathognomonic for Loeffler’s endocarditis as seen in this patient [5,6]. CMR is more sensitive in detecting early changes in cardiac function and structure and help identify changes that may be missed on echocardiography [7]. 

## Conclusions

Loeffler's endocarditis is a cardiac manifestation of hypereosinophilia that can result in diastolic dysfunction or valvulopathy. It is normally seen in patients with HES syndrome. Endomyocardial biopsy is considered as a gold standard; however, it is an invasive procedure. CMR is a noninvasive tool that can help physicians in making an accurate diagnosis.There is no well-defined diagnostic criteria; however, characteristic imaging features can aid in the early detection of the disease and timely therapeutic interventions.
